# (2,2′-Bipyridine-κ^2^ *N*,*N*′)dichloridobis(4-fluoro­benz­yl)tin(IV)

**DOI:** 10.1107/S1600536811015686

**Published:** 2011-05-07

**Authors:** Thy Chun Keng, Kong Mun Lo, Seik Weng Ng

**Affiliations:** a Department of Chemistry, University of Malaya, 50603 Kuala Lumpur, Malaysia

## Abstract

The six-coordinate Sn^IV^ atom in the title compound, [Sn(C_7_H_6_F)_2_Cl_2_(C_10_H_8_N_2_)], shows a *trans*-C_2_SnN_2_Cl_2_ octa­hedral coordination [C—Sn—C = 174.81 (10) and 176.71 (9)° in the two independent mol­ecules in the asymmetric unit]; the Cl atoms are *cis* to each other as are the N atoms of the chelating N-heterocycle.

## Related literature

For the direct synthesis of the organotin chloride reactant, see: Sisido *et al.* (1961 ▶). For the dibenzyl­dichloridotin–2,2′-bipyridine adduct, see: Tiekink *et al.* (2000 ▶).
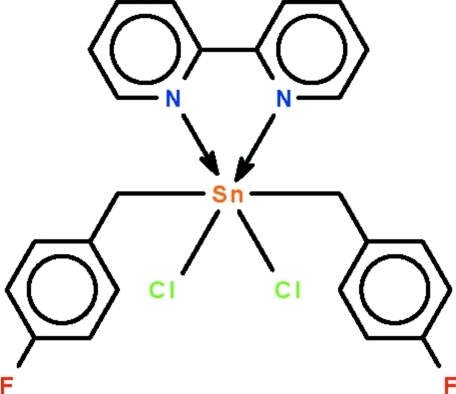


## Experimental

### 

#### Crystal data

[Sn(C_7_H_6_F)_2_Cl_2_(C_10_H_8_N_2_)]
*M* *_r_* = 564.01
Triclinic, 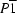

*a* = 7.4918 (1) Å
*b* = 17.4224 (2) Å
*c* = 18.0040 (2) Å
α = 89.9539 (6)°
β = 82.1008 (6)°
γ = 80.2821 (7)°
*V* = 2293.67 (5) Å^3^
*Z* = 4
Mo *K*α radiation
μ = 1.38 mm^−1^
*T* = 100 K
0.30 × 0.25 × 0.20 mm

#### Data collection

Bruker SMART APEX diffractometer
Absorption correction: multi-scan (*SADABS*; Sheldrick, 1996 ▶) *T*_min_ = 0.683, *T*_max_ = 0.770
23316 measured reflections
11247 independent reflections
9206 reflections with *I* > 2σ(*I*)
*R*_int_ = 0.019

#### Refinement

*R*[*F* ^2^ > 2σ(*F* ^2^)] = 0.027
*wR*(*F* ^2^) = 0.073
*S* = 0.95
11247 reflections
559 parameters
H-atom parameters constrained
Δρ_max_ = 0.32 e Å^−3^
Δρ_min_ = −0.49 e Å^−3^

### 

Data collection: *APEX2* (Bruker, 2009 ▶); cell refinement: *SAINT* (Bruker, 2009 ▶); data reduction: *SAINT*; program(s) used to solve structure: *SHELXS97* (Sheldrick, 2008 ▶); program(s) used to refine structure: *SHELXL97* (Sheldrick, 2008 ▶); molecular graphics: *X-SEED* (Barbour, 2001 ▶); software used to prepare material for publication: *publCIF* (Westrip, 2010 ▶).

## Supplementary Material

Crystal structure: contains datablocks global, I. DOI: 10.1107/S1600536811015686/bt5523sup1.cif

Structure factors: contains datablocks I. DOI: 10.1107/S1600536811015686/bt5523Isup2.hkl

Additional supplementary materials:  crystallographic information; 3D view; checkCIF report

## Figures and Tables

**Fig. 1.**
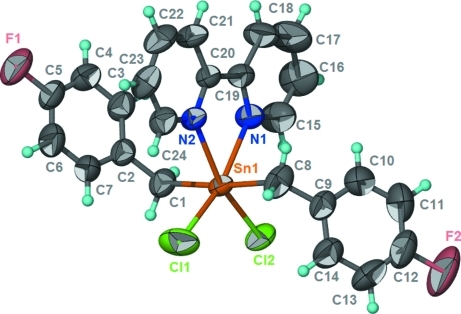
Anisotropic displacement ellipsoid plot (Barbour, 2001) of SnCl2(C10H8N2)(C7H6F)2 at the 70% probability level; hydrogen atoms are drawn as spheres of arbitrary radius.

**Fig. 2.**
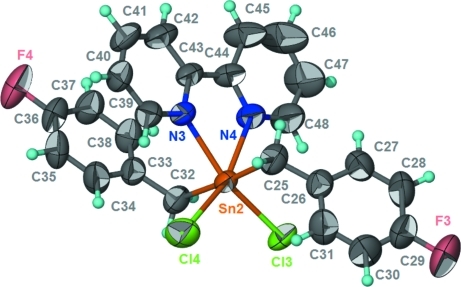
Anisotropic displacement ellipsoid plot (Barbour, 2001) of SnCl2(C10H8N2)(C7H6F)2 at the 70% probability level; hydrogen atoms are drawn as spheres of arbitrary radius.

## supplementary crystallographic information

### Comment

Dibenzyltin dichloride can be synthesized by the direct action of benzyl chloride on tin metal; other ring-substituted analogs are similarly synthesized (Sisido *et al.*, 1961). Dibenzyltin dichloride itself forms a 1:1 adduct with 2,2'-bipyridine (Tiekink *et al.*, 2000). The fluorine-subsituted analog affords the corresponding adduct. The six-coordinate Sn^IV^ atom in SnCl_2_(C_10_H_8_N_2_)(C_7_H_6_F)_2_ (Scheme I) shows *trans*-C_2_SnN_2_Cl_2_ octahedral coordination [C–Sn–C 174.8 (1) °]; the Cl atoms are *cis* to each other as are the N atoms of the chelating *N*-heterocycle.

### Experimental

Di(4-fluorobenzyl)tin dichloride was synthesized by using a literature procedure (Sisido *et al.*, 1961). The compound (0.41 g, 1 mmol) and 2,2'-bipyridine (0156 g, 1 mmol) heated in chloroform (50 ml) until the reactants dissolved completely. The solution was filtered and then set aside for the growth of colorless crystals.

### Refinement

H-atoms were placed in calculated positions (C—H 0.95 to 0.99 Å) and were included in the refinement in the riding model approximation, with *U*(H) set to 1.2 times *U*_eq_(C).

Omitted from the refinement owing to bad agreements were (0 1 0), (3 - 7 5), (2 - 1 11), (8 - 4 13), (2 - 5 20), (-1 19 10) and (2 - 1 12).

### Crystal data

**Table:**

[Sn(C_7_H_6_F)_2_Cl_2_(C_10_H_8_N_2_)]	*Z* = 4
*M**_r_* = 564.01	*F*(000) = 1120
Triclinic, *P*1	*D*_x_ = 1.633 Mg m^−^^3^
Hall symbol: -P 1	Mo *K*α radiation, λ = 0.71073 Å
*a* = 7.4918 (1) Å	Cell parameters from 9696 reflections
*b* = 17.4224 (2) Å	θ = 2.4–28.2°
*c* = 18.0040 (2) Å	µ = 1.38 mm^−^^1^
α = 89.9539 (6)°	*T* = 100 K
β = 82.1008 (6)°	Block, colorless
γ = 80.2821 (7)°	0.30 × 0.25 × 0.20 mm
*V* = 2293.67 (5) Å^3^	

### Data collection

**Table:**

Bruker SMART APEX diffractometer	11247 independent reflections
Radiation source: fine-focus sealed tube	9206 reflections with *I* > 2σ(*I*)
graphite	*R*_int_ = 0.019
ω scans	θ_max_ = 28.3°, θ_min_ = 1.1°
Absorption correction: multi-scan (*SADABS*; Sheldrick, 1996)	*h* = −9→9
*T*_min_ = 0.683, *T*_max_ = 0.770	*k* = −23→23
23316 measured reflections	*l* = −23→23

### Refinement

**Table:**

Refinement on *F*^2^	Primary atom site location: structure-invariant direct methods
Least-squares matrix: full	Secondary atom site location: difference Fourier map
*R*[*F*^2^ > 2σ(*F*^2^)] = 0.027	Hydrogen site location: inferred from neighbouring sites
*wR*(*F*^2^) = 0.073	H-atom parameters constrained
*S* = 0.95	*w* = 1/[σ^2^(*F*_o_^2^) + (0.041*P*)^2^ + 0.3811*P*] where *P* = (*F*_o_^2^ + 2*F*_c_^2^)/3
11247 reflections	(Δ/σ)_max_ = 0.001
559 parameters	Δρ_max_ = 0.32 e Å^−^^3^
0 restraints	Δρ_min_ = −0.49 e Å^−^^3^

### Fractional atomic coordinates and isotropic or equivalent isotropic displacement parameters (Å^2^)

**Table:**

	*x*	*y*	*z*	*U*_iso_*/*U*_eq_	
Sn1	0.50544 (2)	0.735187 (8)	0.230260 (8)	0.03711 (5)	
Sn2	0.95558 (2)	0.252553 (9)	0.265678 (8)	0.03965 (5)	
Cl1	0.70071 (13)	0.62131 (5)	0.15738 (4)	0.0813 (2)	
Cl2	0.41543 (9)	0.82926 (4)	0.13091 (3)	0.05635 (16)	
Cl3	0.88629 (9)	0.13768 (4)	0.34283 (3)	0.05552 (15)	
Cl4	0.76762 (12)	0.36774 (5)	0.33978 (4)	0.0748 (2)	
F1	0.1293 (3)	0.45285 (11)	0.42425 (11)	0.0887 (6)	
F2	0.8851 (4)	1.04628 (14)	0.09035 (16)	0.1353 (10)	
F3	1.3292 (4)	0.07930 (15)	0.56071 (12)	0.1202 (8)	
F4	0.6648 (3)	0.42026 (11)	−0.04628 (10)	0.0934 (6)	
N1	0.3390 (3)	0.81893 (11)	0.32916 (11)	0.0502 (5)	
N2	0.5312 (2)	0.67689 (10)	0.34861 (10)	0.0397 (4)	
N3	1.0714 (3)	0.33062 (11)	0.16923 (11)	0.0453 (4)	
N4	1.1539 (3)	0.17366 (12)	0.17094 (11)	0.0504 (5)	
C1	0.2597 (4)	0.68841 (15)	0.22143 (14)	0.0532 (6)	
H1A	0.1545	0.7318	0.2291	0.064*	
H1B	0.2678	0.6677	0.1697	0.064*	
C2	0.2205 (3)	0.62564 (14)	0.27464 (13)	0.0467 (5)	
C3	0.1175 (3)	0.64260 (15)	0.34415 (15)	0.0550 (6)	
H3	0.0664	0.6953	0.3575	0.066*	
C4	0.0869 (4)	0.58506 (17)	0.39477 (15)	0.0612 (7)	
H4	0.0161	0.5977	0.4425	0.073*	
C5	0.1599 (4)	0.51010 (16)	0.37497 (15)	0.0578 (7)	
C6	0.2613 (5)	0.49040 (16)	0.30735 (18)	0.0760 (9)	
H6	0.3109	0.4375	0.2946	0.091*	
C7	0.2911 (4)	0.54835 (16)	0.25752 (16)	0.0687 (8)	
H7	0.3622	0.5348	0.2101	0.082*	
C8	0.7458 (4)	0.78156 (17)	0.25003 (16)	0.0611 (7)	
H8A	0.7338	0.7942	0.3043	0.073*	
H8B	0.8531	0.7396	0.2383	0.073*	
C9	0.7865 (3)	0.85147 (15)	0.20819 (15)	0.0519 (6)	
C10	0.7426 (5)	0.92446 (19)	0.24093 (18)	0.0787 (9)	
H10	0.6873	0.9301	0.2918	0.094*	
C11	0.7762 (6)	0.9900 (2)	0.2022 (2)	0.0980 (12)	
H11	0.7459	1.0400	0.2260	0.118*	
C12	0.8530 (5)	0.9813 (2)	0.1299 (2)	0.0823 (10)	
C13	0.9025 (5)	0.9116 (2)	0.09500 (19)	0.0855 (10)	
H13	0.9601	0.9071	0.0444	0.103*	
C14	0.8679 (4)	0.84577 (18)	0.13439 (17)	0.0694 (8)	
H14	0.9010	0.7960	0.1101	0.083*	
C15	0.2467 (5)	0.89001 (16)	0.31686 (18)	0.0727 (9)	
H15	0.2354	0.9047	0.2667	0.087*	
C16	0.1684 (5)	0.94192 (19)	0.3739 (2)	0.0924 (12)	
H16	0.1038	0.9917	0.3637	0.111*	
C17	0.1859 (6)	0.9202 (2)	0.4455 (2)	0.0969 (12)	
H17	0.1348	0.9556	0.4860	0.116*	
C18	0.2758 (4)	0.84816 (18)	0.45973 (16)	0.0708 (8)	
H18	0.2860	0.8328	0.5098	0.085*	
C19	0.3521 (3)	0.79757 (14)	0.40011 (13)	0.0464 (5)	
C20	0.4493 (3)	0.71805 (13)	0.41050 (12)	0.0410 (5)	
C21	0.4536 (4)	0.68556 (17)	0.48102 (14)	0.0590 (7)	
H21	0.3958	0.7150	0.5247	0.071*	
C22	0.5423 (4)	0.61029 (17)	0.48685 (16)	0.0660 (8)	
H22	0.5451	0.5872	0.5346	0.079*	
C23	0.6258 (4)	0.56920 (16)	0.42358 (16)	0.0607 (7)	
H23	0.6886	0.5174	0.4266	0.073*	
C24	0.6177 (3)	0.60396 (14)	0.35546 (14)	0.0506 (6)	
H24	0.6758	0.5752	0.3114	0.061*	
C25	1.1941 (4)	0.26824 (16)	0.31589 (15)	0.0587 (7)	
H25A	1.3020	0.2581	0.2766	0.070*	
H25B	1.1789	0.3235	0.3321	0.070*	
C26	1.2348 (3)	0.21893 (15)	0.38120 (14)	0.0510 (6)	
C27	1.3602 (4)	0.15072 (18)	0.37264 (16)	0.0672 (8)	
H27	1.4257	0.1359	0.3244	0.081*	
C28	1.3926 (5)	0.1037 (2)	0.43239 (19)	0.0801 (10)	
H28	1.4789	0.0567	0.4259	0.096*	
C29	1.2988 (5)	0.1259 (2)	0.50054 (18)	0.0755 (9)	
C30	1.1762 (5)	0.1932 (2)	0.51241 (16)	0.0791 (9)	
H30	1.1141	0.2081	0.5612	0.095*	
C31	1.1446 (4)	0.23897 (17)	0.45204 (16)	0.0677 (8)	
H31	1.0580	0.2858	0.4594	0.081*	
C32	0.7243 (4)	0.23876 (16)	0.20922 (14)	0.0553 (6)	
H32A	0.7367	0.1832	0.1944	0.066*	
H32B	0.6114	0.2517	0.2457	0.066*	
C33	0.7006 (3)	0.28644 (14)	0.14155 (14)	0.0483 (5)	
C34	0.6285 (4)	0.36510 (16)	0.14713 (15)	0.0620 (7)	
H34	0.5864	0.3886	0.1953	0.074*	
C35	0.6163 (4)	0.41051 (16)	0.08406 (16)	0.0659 (8)	
H35	0.5678	0.4646	0.0887	0.079*	
C36	0.6751 (4)	0.37585 (16)	0.01597 (15)	0.0602 (7)	
C37	0.7439 (4)	0.29836 (17)	0.00684 (15)	0.0634 (7)	
H37	0.7822	0.2753	−0.0418	0.076*	
C38	0.7563 (4)	0.25428 (15)	0.07055 (15)	0.0565 (6)	
H38	0.8048	0.2002	0.0651	0.068*	
C39	1.0244 (4)	0.40825 (14)	0.16997 (15)	0.0568 (6)	
H39	0.9547	0.4338	0.2138	0.068*	
C40	1.0734 (4)	0.45265 (16)	0.10942 (17)	0.0675 (8)	
H40	1.0400	0.5078	0.1118	0.081*	
C41	1.1705 (4)	0.41574 (18)	0.04632 (16)	0.0684 (8)	
H41	1.2026	0.4448	0.0033	0.082*	
C42	1.2220 (4)	0.33607 (17)	0.04505 (14)	0.0615 (7)	
H42	1.2915	0.3099	0.0015	0.074*	
C43	1.1722 (3)	0.29447 (14)	0.10754 (12)	0.0445 (5)	
C44	1.2293 (3)	0.20931 (15)	0.11152 (13)	0.0504 (6)	
C45	1.3588 (5)	0.16688 (18)	0.05752 (17)	0.0768 (9)	
H45	1.4105	0.1919	0.0149	0.092*	
C46	1.4112 (6)	0.0885 (2)	0.0664 (2)	0.1022 (14)	
H46	1.5029	0.0593	0.0308	0.123*	
C47	1.3321 (5)	0.05250 (19)	0.1259 (2)	0.0923 (12)	
H47	1.3650	−0.0020	0.1318	0.111*	
C48	1.2039 (4)	0.09654 (16)	0.17730 (18)	0.0695 (8)	
H48	1.1482	0.0715	0.2190	0.083*	

### Atomic displacement parameters (Å^2^)

**Table:**

	*U*^11^	*U*^22^	*U*^33^	*U*^12^	*U*^13^	*U*^23^
Sn1	0.03972 (9)	0.03968 (8)	0.02985 (8)	−0.00562 (6)	0.00102 (6)	0.00565 (6)
Sn2	0.04368 (9)	0.04088 (9)	0.03124 (8)	−0.00313 (6)	0.00079 (6)	0.00737 (6)
Cl1	0.0935 (6)	0.0748 (5)	0.0571 (4)	0.0165 (4)	0.0161 (4)	−0.0129 (4)
Cl2	0.0647 (4)	0.0620 (4)	0.0439 (3)	−0.0132 (3)	−0.0099 (3)	0.0221 (3)
Cl3	0.0697 (4)	0.0543 (3)	0.0435 (3)	−0.0169 (3)	−0.0033 (3)	0.0178 (3)
Cl4	0.0879 (5)	0.0648 (4)	0.0562 (4)	0.0099 (4)	0.0161 (4)	−0.0075 (3)
F1	0.1091 (15)	0.0822 (12)	0.0819 (13)	−0.0388 (11)	−0.0115 (11)	0.0396 (10)
F2	0.173 (3)	0.0975 (17)	0.151 (2)	−0.0690 (17)	−0.0228 (19)	0.0648 (16)
F3	0.1342 (19)	0.139 (2)	0.0816 (14)	0.0054 (16)	−0.0292 (13)	0.0552 (14)
F4	0.1475 (18)	0.0764 (12)	0.0650 (11)	−0.0204 (12)	−0.0436 (12)	0.0299 (9)
N1	0.0623 (13)	0.0402 (10)	0.0419 (11)	0.0024 (9)	0.0006 (9)	0.0027 (9)
N2	0.0425 (10)	0.0410 (10)	0.0348 (9)	−0.0058 (8)	−0.0040 (7)	0.0064 (8)
N3	0.0476 (11)	0.0441 (10)	0.0420 (10)	−0.0070 (9)	0.0006 (8)	0.0102 (8)
N4	0.0573 (12)	0.0441 (11)	0.0456 (11)	−0.0065 (9)	0.0051 (9)	0.0017 (9)
C1	0.0586 (15)	0.0588 (15)	0.0490 (14)	−0.0223 (12)	−0.0163 (11)	0.0134 (12)
C2	0.0469 (13)	0.0529 (13)	0.0459 (13)	−0.0205 (11)	−0.0108 (10)	0.0073 (11)
C3	0.0469 (14)	0.0524 (14)	0.0632 (16)	−0.0112 (11)	0.0046 (12)	0.0023 (12)
C4	0.0602 (16)	0.0714 (18)	0.0517 (15)	−0.0221 (14)	0.0072 (12)	0.0021 (13)
C5	0.0664 (17)	0.0592 (16)	0.0540 (15)	−0.0284 (13)	−0.0086 (13)	0.0163 (13)
C6	0.100 (2)	0.0441 (15)	0.081 (2)	−0.0205 (15)	0.0093 (18)	−0.0002 (14)
C7	0.097 (2)	0.0539 (15)	0.0541 (16)	−0.0310 (16)	0.0143 (15)	−0.0074 (13)
C8	0.0490 (15)	0.0706 (17)	0.0696 (18)	−0.0206 (13)	−0.0157 (13)	0.0255 (14)
C9	0.0464 (13)	0.0575 (15)	0.0557 (15)	−0.0194 (11)	−0.0080 (11)	0.0111 (12)
C10	0.102 (3)	0.080 (2)	0.0583 (18)	−0.0394 (19)	0.0021 (17)	−0.0046 (16)
C11	0.137 (4)	0.0589 (19)	0.103 (3)	−0.040 (2)	−0.005 (3)	−0.0041 (19)
C12	0.097 (3)	0.068 (2)	0.091 (3)	−0.0398 (19)	−0.016 (2)	0.0270 (19)
C13	0.091 (2)	0.099 (3)	0.067 (2)	−0.038 (2)	0.0150 (17)	0.0207 (19)
C14	0.0711 (19)	0.0645 (17)	0.0679 (19)	−0.0183 (15)	0.0148 (15)	0.0022 (15)
C15	0.090 (2)	0.0511 (16)	0.0631 (18)	0.0163 (15)	0.0011 (16)	0.0073 (14)
C16	0.109 (3)	0.0525 (17)	0.094 (3)	0.0265 (18)	0.012 (2)	0.0004 (18)
C17	0.116 (3)	0.076 (2)	0.077 (2)	0.021 (2)	0.017 (2)	−0.0212 (19)
C18	0.089 (2)	0.0688 (19)	0.0456 (15)	0.0000 (16)	0.0064 (14)	−0.0094 (14)
C19	0.0511 (13)	0.0477 (13)	0.0383 (12)	−0.0078 (11)	0.0011 (10)	−0.0006 (10)
C20	0.0448 (12)	0.0453 (12)	0.0341 (11)	−0.0127 (10)	−0.0034 (9)	0.0032 (9)
C21	0.0726 (18)	0.0715 (17)	0.0337 (12)	−0.0166 (14)	−0.0047 (11)	0.0105 (12)
C22	0.0768 (19)	0.0751 (19)	0.0488 (15)	−0.0174 (16)	−0.0125 (14)	0.0291 (14)
C23	0.0617 (16)	0.0523 (15)	0.0681 (18)	−0.0073 (12)	−0.0125 (13)	0.0260 (13)
C24	0.0543 (14)	0.0433 (12)	0.0516 (14)	−0.0025 (11)	−0.0053 (11)	0.0085 (11)
C25	0.0613 (16)	0.0608 (16)	0.0592 (16)	−0.0185 (13)	−0.0171 (13)	0.0151 (13)
C26	0.0515 (14)	0.0554 (14)	0.0490 (14)	−0.0119 (12)	−0.0140 (11)	0.0060 (11)
C27	0.0579 (17)	0.079 (2)	0.0567 (16)	0.0085 (15)	−0.0058 (13)	0.0013 (15)
C28	0.076 (2)	0.078 (2)	0.075 (2)	0.0213 (17)	−0.0145 (17)	0.0127 (17)
C29	0.076 (2)	0.091 (2)	0.0600 (18)	−0.0070 (18)	−0.0213 (16)	0.0263 (17)
C30	0.082 (2)	0.107 (3)	0.0416 (15)	0.0043 (19)	−0.0098 (14)	−0.0016 (16)
C31	0.078 (2)	0.0644 (17)	0.0565 (17)	0.0078 (15)	−0.0175 (14)	−0.0077 (14)
C32	0.0529 (15)	0.0641 (16)	0.0518 (14)	−0.0152 (12)	−0.0115 (11)	0.0194 (12)
C33	0.0466 (13)	0.0520 (13)	0.0482 (13)	−0.0087 (11)	−0.0131 (10)	0.0091 (11)
C34	0.0662 (17)	0.0646 (17)	0.0493 (15)	0.0104 (14)	−0.0138 (13)	−0.0016 (13)
C35	0.080 (2)	0.0480 (14)	0.0686 (19)	0.0084 (14)	−0.0300 (16)	0.0058 (13)
C36	0.0785 (19)	0.0576 (15)	0.0508 (15)	−0.0145 (14)	−0.0283 (14)	0.0152 (13)
C37	0.084 (2)	0.0629 (17)	0.0440 (14)	−0.0065 (15)	−0.0188 (14)	0.0002 (13)
C38	0.0714 (18)	0.0448 (13)	0.0556 (15)	−0.0067 (12)	−0.0208 (13)	0.0018 (12)
C39	0.0576 (15)	0.0462 (13)	0.0601 (16)	−0.0007 (11)	0.0044 (12)	0.0132 (12)
C40	0.0666 (18)	0.0544 (15)	0.079 (2)	−0.0060 (13)	−0.0065 (15)	0.0322 (15)
C41	0.077 (2)	0.077 (2)	0.0551 (17)	−0.0254 (16)	−0.0065 (14)	0.0323 (15)
C42	0.0682 (17)	0.0772 (19)	0.0406 (13)	−0.0242 (15)	0.0014 (12)	0.0119 (13)
C43	0.0448 (12)	0.0559 (14)	0.0349 (11)	−0.0158 (11)	−0.0037 (9)	0.0070 (10)
C44	0.0563 (14)	0.0548 (14)	0.0389 (12)	−0.0158 (12)	0.0057 (10)	−0.0016 (11)
C45	0.095 (2)	0.0677 (19)	0.0589 (18)	−0.0222 (17)	0.0294 (16)	−0.0165 (15)
C46	0.117 (3)	0.071 (2)	0.101 (3)	−0.013 (2)	0.045 (2)	−0.034 (2)
C47	0.111 (3)	0.0469 (16)	0.104 (3)	0.0000 (18)	0.021 (2)	−0.0139 (18)
C48	0.087 (2)	0.0448 (14)	0.0677 (19)	−0.0056 (14)	0.0123 (16)	0.0025 (13)

### Geometric parameters (Å, °)

**Table:**

Sn1—C1	2.160 (2)	C17—H17	0.9500
Sn1—C8	2.166 (2)	C18—C19	1.385 (3)
Sn1—N1	2.374 (2)	C18—H18	0.9500
Sn1—N2	2.3775 (17)	C19—C20	1.477 (3)
Sn1—Cl1	2.5090 (7)	C20—C21	1.392 (3)
Sn1—Cl2	2.5089 (6)	C21—C22	1.377 (4)
Sn2—C25	2.167 (3)	C21—H21	0.9500
Sn2—C32	2.172 (3)	C22—C23	1.362 (4)
Sn2—N3	2.3661 (18)	C22—H22	0.9500
Sn2—N4	2.371 (2)	C23—C24	1.372 (3)
Sn2—Cl4	2.5147 (7)	C23—H23	0.9500
Sn2—Cl3	2.5205 (6)	C24—H24	0.9500
F1—C5	1.362 (3)	C25—C26	1.491 (3)
F2—C12	1.375 (3)	C25—H25A	0.9900
F3—C29	1.374 (3)	C25—H25B	0.9900
F4—C36	1.365 (3)	C26—C31	1.375 (4)
N1—C19	1.342 (3)	C26—C27	1.379 (4)
N1—C15	1.347 (3)	C27—C28	1.375 (4)
N2—C24	1.339 (3)	C27—H27	0.9500
N2—C20	1.340 (3)	C28—C29	1.349 (4)
N3—C39	1.338 (3)	C28—H28	0.9500
N3—C43	1.346 (3)	C29—C30	1.359 (4)
N4—C48	1.342 (3)	C30—C31	1.371 (4)
N4—C44	1.342 (3)	C30—H30	0.9500
C1—C2	1.493 (3)	C31—H31	0.9500
C1—H1A	0.9900	C32—C33	1.488 (3)
C1—H1B	0.9900	C32—H32A	0.9900
C2—C7	1.380 (4)	C32—H32B	0.9900
C2—C3	1.381 (3)	C33—C38	1.378 (4)
C3—C4	1.381 (3)	C33—C34	1.384 (4)
C3—H3	0.9500	C34—C35	1.388 (4)
C4—C5	1.356 (4)	C34—H34	0.9500
C4—H4	0.9500	C35—C36	1.351 (4)
C5—C6	1.355 (4)	C35—H35	0.9500
C6—C7	1.376 (4)	C36—C37	1.363 (4)
C6—H6	0.9500	C37—C38	1.385 (4)
C7—H7	0.9500	C37—H37	0.9500
C8—C9	1.485 (3)	C38—H38	0.9500
C8—H8A	0.9900	C39—C40	1.381 (3)
C8—H8B	0.9900	C39—H39	0.9500
C9—C10	1.371 (4)	C40—C41	1.360 (4)
C9—C14	1.380 (4)	C40—H40	0.9500
C10—C11	1.378 (4)	C41—C42	1.376 (4)
C10—H10	0.9500	C41—H41	0.9500
C11—C12	1.347 (5)	C42—C43	1.380 (3)
C11—H11	0.9500	C42—H42	0.9500
C12—C13	1.340 (5)	C43—C44	1.478 (3)
C13—C14	1.390 (4)	C44—C45	1.390 (4)
C13—H13	0.9500	C45—C46	1.371 (5)
C14—H14	0.9500	C45—H45	0.9500
C15—C16	1.368 (4)	C46—C47	1.357 (5)
C15—H15	0.9500	C46—H46	0.9500
C16—C17	1.361 (5)	C47—C48	1.370 (4)
C16—H16	0.9500	C47—H47	0.9500
C17—C18	1.362 (4)	C48—H48	0.9500
			
C1—Sn1—C8	174.81 (10)	C17—C18—C19	119.1 (3)
C1—Sn1—N1	88.70 (9)	C17—C18—H18	120.5
C8—Sn1—N1	87.53 (10)	C19—C18—H18	120.5
C1—Sn1—N2	91.35 (8)	N1—C19—C18	120.8 (2)
C8—Sn1—N2	83.98 (8)	N1—C19—C20	116.6 (2)
N1—Sn1—N2	69.07 (6)	C18—C19—C20	122.6 (2)
C1—Sn1—Cl1	91.60 (8)	N2—C20—C21	120.6 (2)
C8—Sn1—Cl1	90.99 (9)	N2—C20—C19	117.21 (19)
N1—Sn1—Cl1	163.13 (5)	C21—C20—C19	122.2 (2)
N2—Sn1—Cl1	94.06 (5)	C22—C21—C20	119.4 (3)
C1—Sn1—Cl2	87.44 (6)	C22—C21—H21	120.3
C8—Sn1—Cl2	96.31 (7)	C20—C21—H21	120.3
N1—Sn1—Cl2	93.10 (5)	C23—C22—C21	119.4 (2)
N2—Sn1—Cl2	162.16 (5)	C23—C22—H22	120.3
Cl1—Sn1—Cl2	103.76 (3)	C21—C22—H22	120.3
C25—Sn2—C32	176.71 (9)	C22—C23—C24	118.8 (3)
C25—Sn2—N3	84.58 (8)	C22—C23—H23	120.6
C32—Sn2—N3	92.30 (8)	C24—C23—H23	120.6
C25—Sn2—N4	88.26 (10)	N2—C24—C23	122.6 (2)
C32—Sn2—N4	89.65 (9)	N2—C24—H24	118.7
N3—Sn2—N4	69.52 (7)	C23—C24—H24	118.7
C25—Sn2—Cl4	90.70 (8)	C26—C25—Sn2	116.76 (17)
C32—Sn2—Cl4	90.53 (8)	C26—C25—H25A	108.1
N3—Sn2—Cl4	93.32 (5)	Sn2—C25—H25A	108.1
N4—Sn2—Cl4	162.83 (5)	C26—C25—H25B	108.1
C25—Sn2—Cl3	96.00 (7)	Sn2—C25—H25B	108.1
C32—Sn2—Cl3	86.67 (7)	H25A—C25—H25B	107.3
N3—Sn2—Cl3	163.00 (5)	C31—C26—C27	117.6 (2)
N4—Sn2—Cl3	93.49 (5)	C31—C26—C25	120.9 (2)
Cl4—Sn2—Cl3	103.65 (2)	C27—C26—C25	121.5 (2)
C19—N1—C15	118.8 (2)	C28—C27—C26	121.5 (3)
C19—N1—Sn1	118.45 (15)	C28—C27—H27	119.3
C15—N1—Sn1	122.43 (18)	C26—C27—H27	119.3
C24—N2—C20	119.1 (2)	C29—C28—C27	118.4 (3)
C24—N2—Sn1	122.63 (16)	C29—C28—H28	120.8
C20—N2—Sn1	118.21 (14)	C27—C28—H28	120.8
C39—N3—C43	118.8 (2)	C28—C29—C30	122.6 (3)
C39—N3—Sn2	122.73 (16)	C28—C29—F3	119.0 (3)
C43—N3—Sn2	118.01 (15)	C30—C29—F3	118.4 (3)
C48—N4—C44	119.2 (2)	C29—C30—C31	118.1 (3)
C48—N4—Sn2	122.75 (18)	C29—C30—H30	120.9
C44—N4—Sn2	117.81 (16)	C31—C30—H30	120.9
C2—C1—Sn1	116.18 (16)	C30—C31—C26	121.8 (3)
C2—C1—H1A	108.2	C30—C31—H31	119.1
Sn1—C1—H1A	108.2	C26—C31—H31	119.1
C2—C1—H1B	108.2	C33—C32—Sn2	116.15 (17)
Sn1—C1—H1B	108.2	C33—C32—H32A	108.2
H1A—C1—H1B	107.4	Sn2—C32—H32A	108.2
C7—C2—C3	117.1 (2)	C33—C32—H32B	108.2
C7—C2—C1	121.4 (2)	Sn2—C32—H32B	108.2
C3—C2—C1	121.5 (2)	H32A—C32—H32B	107.4
C2—C3—C4	121.8 (2)	C38—C33—C34	117.3 (2)
C2—C3—H3	119.1	C38—C33—C32	120.9 (2)
C4—C3—H3	119.1	C34—C33—C32	121.7 (2)
C5—C4—C3	118.6 (2)	C33—C34—C35	121.7 (3)
C5—C4—H4	120.7	C33—C34—H34	119.2
C3—C4—H4	120.7	C35—C34—H34	119.2
C6—C5—C4	121.9 (2)	C36—C35—C34	118.3 (3)
C6—C5—F1	119.0 (3)	C36—C35—H35	120.9
C4—C5—F1	119.1 (3)	C34—C35—H35	120.9
C5—C6—C7	118.9 (3)	C35—C36—C37	122.7 (2)
C5—C6—H6	120.6	C35—C36—F4	118.6 (3)
C7—C6—H6	120.6	C37—C36—F4	118.6 (3)
C6—C7—C2	121.8 (3)	C36—C37—C38	118.0 (3)
C6—C7—H7	119.1	C36—C37—H37	121.0
C2—C7—H7	119.1	C38—C37—H37	121.0
C9—C8—Sn1	117.90 (17)	C33—C38—C37	122.0 (2)
C9—C8—H8A	107.8	C33—C38—H38	119.0
Sn1—C8—H8A	107.8	C37—C38—H38	119.0
C9—C8—H8B	107.8	N3—C39—C40	122.5 (3)
Sn1—C8—H8B	107.8	N3—C39—H39	118.8
H8A—C8—H8B	107.2	C40—C39—H39	118.8
C10—C9—C14	117.2 (2)	C41—C40—C39	118.5 (3)
C10—C9—C8	121.4 (3)	C41—C40—H40	120.7
C14—C9—C8	121.4 (3)	C39—C40—H40	120.7
C9—C10—C11	122.0 (3)	C40—C41—C42	119.7 (2)
C9—C10—H10	119.0	C40—C41—H41	120.2
C11—C10—H10	119.0	C42—C41—H41	120.2
C12—C11—C10	118.4 (3)	C41—C42—C43	119.5 (3)
C12—C11—H11	120.8	C41—C42—H42	120.2
C10—C11—H11	120.8	C43—C42—H42	120.2
C13—C12—C11	122.6 (3)	N3—C43—C42	120.9 (2)
C13—C12—F2	118.3 (3)	N3—C43—C44	116.50 (19)
C11—C12—F2	119.0 (4)	C42—C43—C44	122.6 (2)
C12—C13—C14	118.6 (3)	N4—C44—C45	120.3 (2)
C12—C13—H13	120.7	N4—C44—C43	117.3 (2)
C14—C13—H13	120.7	C45—C44—C43	122.4 (2)
C9—C14—C13	121.2 (3)	C46—C45—C44	119.3 (3)
C9—C14—H14	119.4	C46—C45—H45	120.4
C13—C14—H14	119.4	C44—C45—H45	120.4
N1—C15—C16	122.5 (3)	C47—C46—C45	120.1 (3)
N1—C15—H15	118.7	C47—C46—H46	119.9
C16—C15—H15	118.7	C45—C46—H46	119.9
C17—C16—C15	118.1 (3)	C46—C47—C48	118.5 (3)
C17—C16—H16	120.9	C46—C47—H47	120.7
C15—C16—H16	120.9	C48—C47—H47	120.7
C16—C17—C18	120.6 (3)	N4—C48—C47	122.5 (3)
C16—C17—H17	119.7	N4—C48—H48	118.8
C18—C17—H17	119.7	C47—C48—H48	118.8
			
C1—Sn1—N1—C19	97.58 (19)	Sn1—N1—C19—C18	172.3 (2)
C8—Sn1—N1—C19	−78.87 (19)	C15—N1—C19—C20	177.9 (2)
N2—Sn1—N1—C19	5.63 (17)	Sn1—N1—C19—C20	−8.3 (3)
Cl1—Sn1—N1—C19	6.4 (3)	C17—C18—C19—N1	0.3 (5)
Cl2—Sn1—N1—C19	−175.06 (18)	C17—C18—C19—C20	−179.0 (3)
C1—Sn1—N1—C15	−88.9 (2)	C24—N2—C20—C21	−0.2 (3)
C8—Sn1—N1—C15	94.6 (2)	Sn1—N2—C20—C21	177.31 (18)
N2—Sn1—N1—C15	179.1 (3)	C24—N2—C20—C19	−178.7 (2)
Cl1—Sn1—N1—C15	179.9 (2)	Sn1—N2—C20—C19	−1.2 (3)
Cl2—Sn1—N1—C15	−1.6 (2)	N1—C19—C20—N2	6.3 (3)
C1—Sn1—N2—C24	87.19 (19)	C18—C19—C20—N2	−174.3 (2)
C8—Sn1—N2—C24	−95.1 (2)	N1—C19—C20—C21	−172.2 (2)
N1—Sn1—N2—C24	175.3 (2)	C18—C19—C20—C21	7.2 (4)
Cl1—Sn1—N2—C24	−4.51 (18)	N2—C20—C21—C22	−0.2 (4)
Cl2—Sn1—N2—C24	173.05 (14)	C19—C20—C21—C22	178.2 (2)
C1—Sn1—N2—C20	−90.19 (17)	C20—C21—C22—C23	0.6 (4)
C8—Sn1—N2—C20	87.55 (18)	C21—C22—C23—C24	−0.6 (4)
N1—Sn1—N2—C20	−2.10 (15)	C20—N2—C24—C23	0.2 (4)
Cl1—Sn1—N2—C20	178.11 (16)	Sn1—N2—C24—C23	−177.15 (19)
Cl2—Sn1—N2—C20	−4.3 (3)	C22—C23—C24—N2	0.2 (4)
C25—Sn2—N3—C39	−91.0 (2)	N3—Sn2—C25—C26	−169.4 (2)
C32—Sn2—N3—C39	90.0 (2)	N4—Sn2—C25—C26	−99.8 (2)
N4—Sn2—N3—C39	178.8 (2)	Cl4—Sn2—C25—C26	97.3 (2)
Cl4—Sn2—N3—C39	−0.6 (2)	Cl3—Sn2—C25—C26	−6.5 (2)
Cl3—Sn2—N3—C39	176.18 (16)	Sn2—C25—C26—C31	−81.3 (3)
C25—Sn2—N3—C43	96.52 (18)	Sn2—C25—C26—C27	96.9 (3)
C32—Sn2—N3—C43	−82.42 (18)	C31—C26—C27—C28	0.9 (5)
N4—Sn2—N3—C43	6.35 (16)	C25—C26—C27—C28	−177.4 (3)
Cl4—Sn2—N3—C43	−173.09 (16)	C26—C27—C28—C29	−0.2 (5)
Cl3—Sn2—N3—C43	3.7 (3)	C27—C28—C29—C30	−1.0 (6)
C25—Sn2—N4—C48	89.0 (2)	C27—C28—C29—F3	179.7 (3)
C32—Sn2—N4—C48	−93.6 (2)	C28—C29—C30—C31	1.5 (6)
N3—Sn2—N4—C48	173.9 (2)	F3—C29—C30—C31	−179.2 (3)
Cl4—Sn2—N4—C48	175.75 (19)	C29—C30—C31—C26	−0.9 (5)
Cl3—Sn2—N4—C48	−6.9 (2)	C27—C26—C31—C30	−0.3 (5)
C25—Sn2—N4—C44	−85.7 (2)	C25—C26—C31—C30	178.0 (3)
C32—Sn2—N4—C44	91.7 (2)	N3—Sn2—C32—C33	−7.6 (2)
N3—Sn2—N4—C44	−0.86 (18)	N4—Sn2—C32—C33	−77.1 (2)
Cl4—Sn2—N4—C44	1.0 (3)	Cl4—Sn2—C32—C33	85.7 (2)
Cl3—Sn2—N4—C44	178.37 (18)	Cl3—Sn2—C32—C33	−170.6 (2)
N1—Sn1—C1—C2	−83.5 (2)	Sn2—C32—C33—C38	100.6 (3)
N2—Sn1—C1—C2	−14.4 (2)	Sn2—C32—C33—C34	−76.9 (3)
Cl1—Sn1—C1—C2	79.7 (2)	C38—C33—C34—C35	−1.4 (4)
Cl2—Sn1—C1—C2	−176.6 (2)	C32—C33—C34—C35	176.2 (3)
Sn1—C1—C2—C7	−87.5 (3)	C33—C34—C35—C36	0.7 (5)
Sn1—C1—C2—C3	90.2 (3)	C34—C35—C36—C37	0.6 (5)
C7—C2—C3—C4	0.4 (4)	C34—C35—C36—F4	−179.5 (3)
C1—C2—C3—C4	−177.4 (2)	C35—C36—C37—C38	−1.2 (5)
C2—C3—C4—C5	−0.3 (4)	F4—C36—C37—C38	178.9 (3)
C3—C4—C5—C6	0.0 (5)	C34—C33—C38—C37	0.8 (4)
C3—C4—C5—F1	−179.5 (2)	C32—C33—C38—C37	−176.8 (2)
C4—C5—C6—C7	0.2 (5)	C36—C37—C38—C33	0.4 (4)
F1—C5—C6—C7	179.7 (3)	C43—N3—C39—C40	0.9 (4)
C5—C6—C7—C2	0.0 (5)	Sn2—N3—C39—C40	−171.5 (2)
C3—C2—C7—C6	−0.2 (4)	N3—C39—C40—C41	1.1 (5)
C1—C2—C7—C6	177.6 (3)	C39—C40—C41—C42	−2.0 (5)
N1—Sn1—C8—C9	−89.8 (2)	C40—C41—C42—C43	0.9 (4)
N2—Sn1—C8—C9	−159.1 (2)	C39—N3—C43—C42	−2.0 (4)
Cl1—Sn1—C8—C9	107.0 (2)	Sn2—N3—C43—C42	170.81 (18)
Cl2—Sn1—C8—C9	3.0 (2)	C39—N3—C43—C44	176.5 (2)
Sn1—C8—C9—C10	100.1 (3)	Sn2—N3—C43—C44	−10.8 (3)
Sn1—C8—C9—C14	−79.1 (3)	C41—C42—C43—N3	1.1 (4)
C14—C9—C10—C11	0.5 (5)	C41—C42—C43—C44	−177.2 (2)
C8—C9—C10—C11	−178.7 (3)	C48—N4—C44—C45	−0.5 (4)
C9—C10—C11—C12	0.6 (6)	Sn2—N4—C44—C45	174.4 (2)
C10—C11—C12—C13	−2.0 (7)	C48—N4—C44—C43	−179.1 (2)
C10—C11—C12—F2	179.2 (3)	Sn2—N4—C44—C43	−4.2 (3)
C11—C12—C13—C14	2.1 (6)	N3—C43—C44—N4	9.9 (3)
F2—C12—C13—C14	−179.1 (3)	C42—C43—C44—N4	−171.7 (2)
C10—C9—C14—C13	−0.5 (5)	N3—C43—C44—C45	−168.6 (3)
C8—C9—C14—C13	178.7 (3)	C42—C43—C44—C45	9.8 (4)
C12—C13—C14—C9	−0.8 (5)	N4—C44—C45—C46	−1.2 (5)
C19—N1—C15—C16	1.2 (5)	C43—C44—C45—C46	177.2 (3)
Sn1—N1—C15—C16	−172.2 (3)	C44—C45—C46—C47	2.4 (6)
N1—C15—C16—C17	0.1 (6)	C45—C46—C47—C48	−1.8 (7)
C15—C16—C17—C18	−1.2 (7)	C44—N4—C48—C47	1.2 (5)
C16—C17—C18—C19	1.0 (6)	Sn2—N4—C48—C47	−173.4 (3)
C15—N1—C19—C18	−1.4 (4)	C46—C47—C48—N4	0.0 (6)
